# The Annual Meeting of the American Public Health Association

**Published:** 1881-01

**Authors:** 


THE ANNUAL MEETING OF THE AMERICAN PUBLIC HEALTH ASSOCIATION took place in New Orleans on the 7th, 8th, 9th and 10th of December, last, and about five hundred members were present. Several very good papers were read and their subjects discussed. The next meeting will be held next year in Savannah, Georgia.

